# Contained rupture of arch aneurysm managed with total arch, frozen elephant trunk and endograft

**DOI:** 10.21542/gcsp.2019.9

**Published:** 2019-03-31

**Authors:** Michael Ibrahim, Roland Assi, Fenton McCarthy, Michael A. Golden, Wilson Y Szeto

**Affiliations:** Division of Cardiovascular Surgery, University of Pennsylvania, Penn Presbyterian Medical Center, 51 North 39^th^ Street, Heart and Vascular Pavilion Suite 2A, Philadelphia PA 19104, USA

## Abstract

We here describe a complex case of a 75-year-old man presenting with contained rupture of an aortic arch aneurysm in the presence of a second thoracic aortic aneurysm. He was managed with emergent total arch replacement with frozen elephant trunk. Another stent-graft was used to achieve hemostasis at the distal anastomosis. He later underwent TEVAR extension to manage his second aneurysm in a staged fashion. This case demonstrates a number of important concepts in the evolving interaction between open and endovascular therapies of the aortic arch, particularly in the emergent setting.

## Introduction

Contained rupture of an aortic arch aneurysm is one of the most devastating and complex problems faced by cardiac surgeons with significant morbidity and mortality^1^. Endovascular approaches to the aortic arch are in evolution, with the advent of arch branched graft technology which can be tailor made to manage specific aneurysm and arch morphology^2–4^, as off-the-shelf devices may be inadequate to provide a single endovascular solution. In the case of patients presenting urgently however, there may not be time to allow for tailor made solutions. In this setting, hybrid procedures with adaptive use of ready-made devices may be the optimal solution.

**Figure 1.**
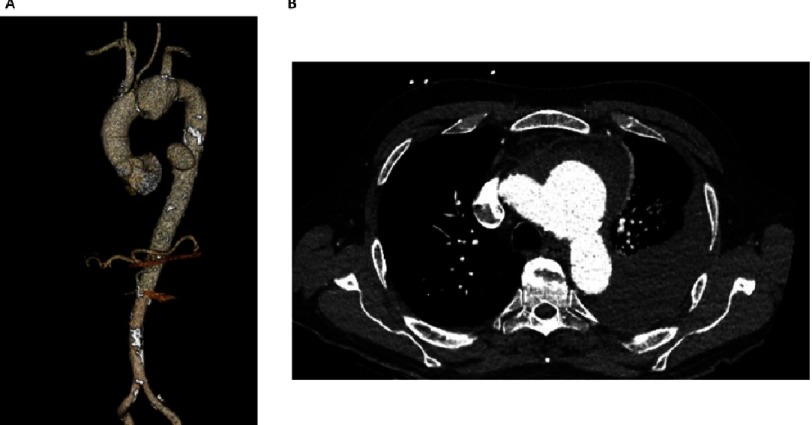
Pre-operative CT angiography of the thoracic aorta. (A) Between the origins of the left common carotid and left subclavian arteries (LSCA), there was a 3.7 × 4.4 × 5.2 cm aneurysm directed towards the left, inferiorly and posteriorly. A second 4.3 cm saccular aneurysm arose from left lateral wall of the descending thoracic aorta. (B) The presence of peri-aneurysmal fluid, left hemothorax, and hemopericardium as well as his clinical presentation, confirmed relatively acute contained rupture (Figure 1B).

## Case report

We present the case of a 75-year-old man with a history of hyperlipidemia, giant cell arteritis on steroids, and recent urinary tract infection on antibiotics, who presented acutely with a complex aortic arch aneurysm. Ten days prior, he experienced chest pain and voice hoarseness, which persisted. CT angiogram revealed two saccular aortic aneurysms arising from penetrating atherosclerotic ulcers (PAUs). Between the origins of the left common carotid and left subclavian arteries (LSCA), there was a 3.7 × 4.4 × 5.2 cm aneurysm directed towards the left, inferiorly and posteriorly (Figure 1A). A second 4.3 cm saccular aneurysm arose from left lateral wall of the descending thoracic aorta. The presence of peri-aneurysmal fluid, left hemothorax, and hemopericardium as well as his clinical presentation, confirmed relatively acute contained rupture (Figure 1B). The patient was hemodynamically stable, with weak but symmetrical peripheral pulses and a normal neurologic exam except for a hoarse voice. We counselled him about the natural history and possible therapies including medical management, possible enrollment in the Terumo Aortic Relay Plus Dual Branched-graft clinical trial (NCT03214601) or open therapy. Over the following 6 hours, the patient became hypotensive with a drop in his hemoglobin concentration, so with the patient’s consent, we proceeded emergently to the hybrid operating room.

**Figure 2.**
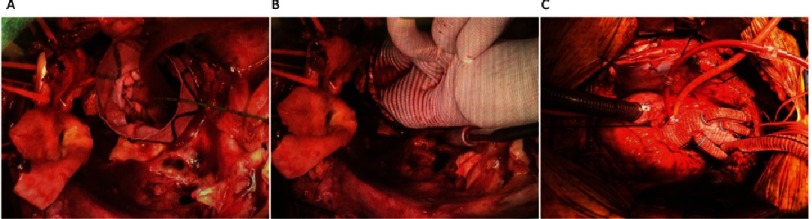
Operative Sequence. (A) First a TEVAR was deployed retrograde from the femoral artery through to the thoracic aorta with its bare Dacron end protruding from the cut edge of the distal aorta. (B) A trifurcated arch graft was sown to the cut edge of the distal aortic arch and sequentially the LSCA, LCC and innominate arteries were anastomsed. (C) The final result showing total arch replacement.

**Figure 3.**
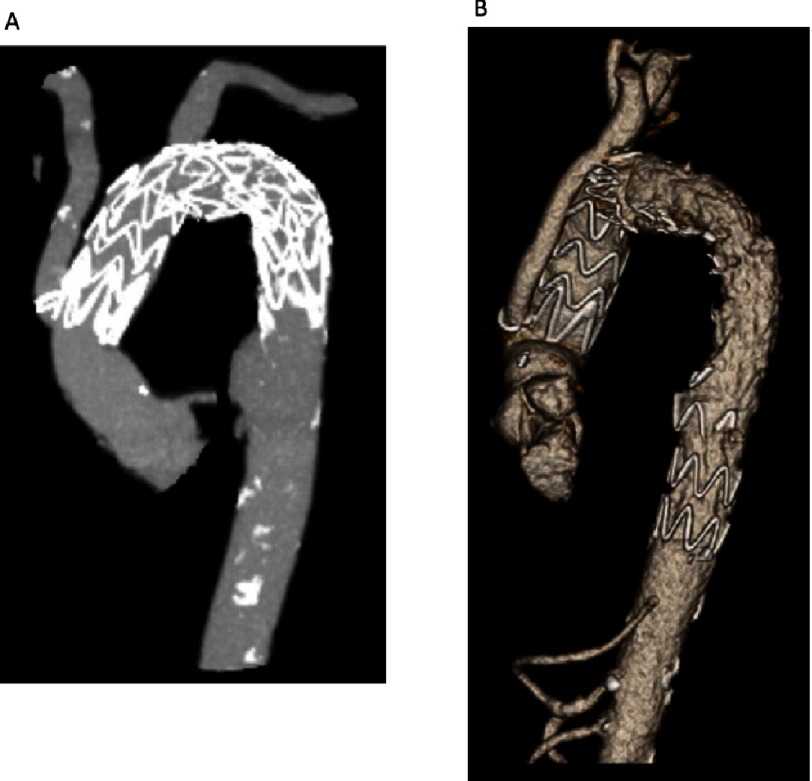
Post-operative CT angiography of the thoracic aorta. (A) Interval CT angiogram re-demonstrated the second saccular aneurysm which had now grown to 2.9 × 3.8 cm, as well as two PAUs. The patient was offered endovascular repair which we performed 13 weeks after the index procedure. (B) He underwent TEVAR extension with 36 × 200 mm TerumoAortic Relay Plus endograft with use of a spinal drain. Completion angiogram revealed no endoleak and the final CT shows exclusion of the second aneurysm and PAUs.

Right axillary cannulation was performed for systemic and later antegrade cerebral perfusion (ACP), by suturing a 10 mm Dacron graft to the right axillary artery. A right femoral arterial sheath was placed through which a soft guidewire was traversed to the descending thoracic aorta under transesophageal echocardiographic (TEE) guidance. Following sternotomy, the patient was placed on cardiopulmonary bypass (CPB) and cooling was started to 28 °C. The aorta was cross-clamped and dissected. On inspection, the ascending aorta had heavy intramural hematoma requiring debridement to the sinotubular junction, which was reconstructed with felt. The aortic valve was structurally normal and was resuspended at the level of the commissures. Upon reaching 28 °C, hypothermic circulatory arrest (HCA) was initiated with ACP through the right axillary artery. Extensive destruction of the distal aortic arch including the os of the LSCA, necessitated debridement to zone 3. The cut end of the proximal DTA was inflamed, atherosclerotic, and friable. Next, a TEVAR (34 × 100 mm Terumo Aortic Relay Plus) was deployed antegrade over the guidewire placed initially through the right femoral artery (Figure 2A). The stent-graft size was based on measurements on the CT scan (oversizing by approximately 10%). Use of the TEVAR as a frozen elephant trunk (FET), allowed reconstruction of this totally destroyed arch tissue, and provided a stable platform on which to construct the proximal arch graft, as well as a landing zone for future TEVAR within the FET. Importantly, when deployed antegrade, the Terumo Aortic graft allows a Dacron end to sew to. The proximal end of the TEVAR stent-graft was allowed to protrude 1.5 cm out of the arch. The cut end of the DTA was approximated to the stent-graft using a running horizontal 4-0 polypropylene suture. The distal anastomosis was constructed between the stent-graft and a 30 × 10 × 8 × 8 mm trifurcated Dacron arch graft (Figure 2B). CPB was resumed and the arch vessels were anastomosed starting with the LSCA, with sequential replacement of the clamp proximal to each completed anastomosis. During this time, the body was perfused with CPB and the brain with ACP for a total of 55 minutes. Finally, the graft to STJ anastomosis was completed (Figure 2C). Cross clamp was removed after 189 minutes. There was significant bleeding at the stent-Dacron graft anastomosis due to the stiff nature of the stent-graft which did not conform well to the Dacron graft to allow for hemostasis. Therefore, a second proximal retrograde TEVAR (Terumo Aortic 36 X 150 mm) was deployed via right femoral artery under fluoroscopic guidance. The proximal landing zone was at the level of the proximal arch just distal to the take-off of the side branches. The patient was weaned from CPB with ease after a total of 258 minutes, hemostasis was achieved and ultrasound examination revealed bilateral carotid flow. The patient’s postoperative recovery was complicated by poor calorie intake requiring the placement of a feeding gastrostomy and a perforated stress gastric ulcer that required emergent exploratory laparotomy and repair. He also required tracheostomy for airway protection due to weakness and vocal cord paralysis. He was subsequently discharged to a rehabilitation facility on post-operative day 14. In follow-up, the patient was clinically well and resumed light activities. Interval CT angiogram re-demonstrated the second saccular aneurysm which had now grown to 2.9 x 3.8 cm, as well as two PAUs (Figure 3A). The patient was offered endovascular repair which we performed 13 weeks after the index procedure. He underwent TEVAR extension with 36 X 200 mm Terumo Aortic Relay Plus endograft with use of a spinal drain. Completion angiogram revealed no endoleak and the final CT shows exclusion of the second aneurysm and PAUs (Figure 3B). The patient was discharged in excellent condition on lifelong suppressive antibiotics for Salmonella UTI in the setting of extensive graft material. No definitive evidence of salmonella aortitis was found.

## Comment

This case demonstrates a number of important points. First, the multiple aneurysms, extensive rupture and multiple PAUs in an elderly but fit patient demonstrates the current clinical equipoise between established open operations and emerging branch graft technology. In the future, such pathology may be addressed endovascularly^5^. Second, hybrid approaches can be deployed in innovative ways to assist in open reconstruction, as the arch tissue in this case would not support a distal anastomosis without the additional support of a FET; in addition, that FET became a support for a second stage endovascular repair. This emphasizes the point that open aortic repairs are now approached in anticipation of facilitating subsequent endovascular therapy. Third, this case demonstrates the dynamic nature of complex aortopathy. On the initial CT available, the ascending aorta showed minimal intramural hematoma, and debranching could have facilitated use of the ascending and proximal arch as a proximal landing zone; however, by the time the patient reached the operating room, the ascending aorta showed extensive destructive IMH necessitating debridement. Fourth, a staged approach to treatment of the second aneurysm is important in that it allows for time for renal recovery prior to additional contrast and may theoretically reduce the risk of spinal ischemia compared to a single stage approach^6,7^.

In summary, this case highlights the current cross roads of endovascular therapy which is facilitating open repair, laying the groundwork for subsequent endovascular solutions and when mature may replace open repair in the high-risk patient.
